# Differential glatiramer acetate treatment persistence in treatment-naive patients compared to patients previously treated with interferon

**DOI:** 10.1186/s12883-015-0399-9

**Published:** 2015-08-19

**Authors:** Mireya Fernández-Fournier, Antonio Tallón-Barranco, Beatriz Chamorro, Patricia Martínez-Sánchez, Inmaculada Puertas

**Affiliations:** Clinical Neuroimmunology and Multiple Sclerosis Unit, La Paz University Hospital, Madrid, Spain; Department of Neurology, La Paz University Hospital, Madrid, Spain

## Abstract

**Background:**

In the treatment of multiple sclerosis, a change of therapy is considered after treatment failure or adverse events. Although disease modifying drugs’ (DMD) efficacy and side effects have been fully analysed in clinical trials, the effects of previous therapy use are less well studied. We aimed to study medication persistence with glatiramer acetate in treatment-naive patients and in patients previously treated with interferon.

**Methods:**

A retrospective study of relapsing-remitting multiple sclerosis patients treated with glatiramer acetate in an MS Unit of a Spanish University Hospital (January 2004 – September 2013). Treatment time on glatiramer acetate was studied. Reasons for treatment discontinuation were considered as follows: lack of efficacy, serious adverse event, injection-related side effect, pregnancy and lost to follow-up. Use of prior DMD was registered and analysed. Homogeneity of groups was analysed using Fisher's and Mann-Whitney’s tests. The Kaplan Meier method and Cox regression model were used to estimate time to and risk of treatment discontinuation.

**Results:**

In total, 155 relapsing-remitting multiple sclerosis patients were treated with glatiramer acetate: 100 treatment-naive patients and 55 treated previously with interferon. At the end of the study, 76 patients (49.0 %) continued on glatiramer acetate (with an average treatment time (ATT) of 50.4 months, s.d.32.8) and 50 patients (32.3 %) had switched therapy: 27 patients (17.4 %) for inefficacy (ATT 29.2 months, s.d.17.5), 20 patients (12.9 %) for injection site reactions (ATT 16.5 months, s.d.20.3) and 3 patients (1.9 %) after serious adverse events (ATT 15.7 months, s.d.15.1). ATT in our cohort was 39 months (s.d.30.0), median follow-up 34 months. Six months after glatiramer acetate initiation, probability of persisting on GA was 91.4 %, 82.5 % after 12 months and 72.5 % after 2 years. The risk of glatiramer acetate treatment discontinuation was 2.8 [1.7 – 4.8] times greater for treatment-naive patients than for patients treated previously with interferon and this was hardly modified after adjusting for sex and age.

**Conclusions:**

Glatiramer acetate was safe and useful with low rates of serious adverse events and low rates of break-through disease. Injection intolerance proved a major limitation to glatiramer acetate use. Patients who had been previously treated with interferons presented a lower probability of glatiramer acetate discontinuation than treatment-naive patients.

## Background

When treating multiple sclerosis (MS) patients, the main goal is to slow disease progression and improve health-related quality of life [1]. Selecting an optimal individualized treatment for MS patients is becoming progressively more complicated as treatment options and their side effects increase [2, 3].

Both interferons (IFNs) and glatiramer acetate (GA) are injectable disease modifying drugs (DMDs) approved and used as first-line therapies in MS. If disease activity is still observed despite treatment or if adverse events occur, a therapy switch is considered. Some studies conclude that switching between injectable DMDs, mainly from IFNs to GA, can result in a substantial relapse rate reduction, both for patients switched for treatment failure as well as for those who are switched for other reasons [4–8].

Although DMD efficacy and side effects have been fully studied in clinical trials, the effects of previous therapy use are less well known. Analysing experience gathered from ‘real-world’ clinical practice could help make rational individualised treatment decisions. Treatment persistence with GA when used after treatment failure with IFNs is not well characterized. The aim of this pilot exploratory study was to describe GA use and treatment persistence, measured as time to GA treatment discontinuation, in a group of Relapsing Remitting MS (RRMS) treatment-naive patients and in a group of RRMS patients who had previously received treatment with IFNs.

## Methods

### Study design

An independent, retrospective observational study was conducted at an MS Unit of a tertiary University Hospital. Clinical records were used to identify patients who had been prescribed glatiramer acetate (GA) between January 1st 2004 and October 1st 2013. Clinical diagnosis, prior DMD treatment, dates of GA treatment initiation and end, as well as reasons for GA treatment discontinuation, were studied. Patients were included according to their clinical history in one of the following groups: treatment-naive patients and patients treated previously with IFN. All RRMS patients who had been exposed to IFN before initiating treatment with GA were included.

Treatment failure was defined as reasons given by the treating neurologist for GA discontinuation. The following reasons were considered: lack of efficacy (including relapses, progression of disability or magnetic resonance imaging (MRI) activity), occurrence of serious adverse event (SAE), injection site reactions, pregnancy or pregnancy planning at study end-point and loss to follow-up. Time to treatment discontinuation was studied. End of observation period was October 1st 2013. Treatment time for each individual patient was calculated in months as the time lapse between GA treatment initiation and the last documented injection. Average treatment times (ATT) were calculated for each group.

### Patients

The study was conducted at the Neurology Department of La Paz University Hospital in Madrid. In total 155 patients met inclusion criteria: age ≥18 years, RRMS diagnosis according to 2010 revised McDonald criteria [9] and treatment with GA between January 1^st^ 2004 - October 1^st^ 2013. Patients were excluded if another diagnosis different from MS was given on follow-up. Patients were then excluded from statistical analysis if data regarding GA initiation and end dates was missing.

Throughout the study period DMD initiation and change were performed at the discretion of the MS Unit medical team following usual clinical practice [3, 10, 11]. A lack of efficacy or suboptimal response to GA was considered the reason for a treatment change based on the patient’s clinical history, considering the occurrence of relapses (≥2 relapses or 1 severe relapse), accrual of disability or disease activity on follow-up MRI (presence of gadolinium-enhancing lesions or increased T2-lesion load, including both new and enlarging T2-lesions). Clinical, including EDSS scores, DMD initiation and end dates, and MRI data were collected and stored in an electronic database. All patients underwent regular follow-up consisting of six-monthly neurological assessments and laboratory testing including liver and thyroid gland function. After therapy approval by the health authorities, all patients received training sessions on self-injectable DMD administration by a nurse who specialised in the care of MS patients. In the training sessions realistic expectations were established, side effects were anticipated, as well as their management, and administration technique was taught. After training, patients self-injected the DMD at home. If they had questions, patients could use a telephone help-line staffed by nurses and neurologists. Unscheduled visits were performed for the assessment and treatment of side effects, relapses or any other clinically relevant condition. This study is a retrospective analysis of our MS clinical data, and has been conducted using data that was anonymized for analysis so specific written informed consent was not obtained from patients. This study has been performed according to the regulations of the Ethics Committee of La Paz University Hospital.

### Data analysis

Statistical analysis was performed using SAS 93 (SAS Institute, Cary, NC, USA). Quantitative data are described using the mean ± standard deviation (± s.d.), median and range. Qualitative data are described using absolute frequencies and percentages. The homogeneity of the groups was analysed using Fisher's exact test for categorical data and Mann-Whitney’s test for quantitative data. Treatment time on GA was studied. The Kaplan Meier method was used to estimate time to treatment discontinuation. Risk of treatment discontinuation was estimated using a Cox regression model, with and without adjusting for sex and age at the time of GA initiation. The independent variable of this study was time to GA treatment discontinuation, defined as the time lapse between treatment initiation and treatment discontinuation in months. Patients who were still treated with GA on October 1st 2013 and those lost to follow up on treatment with GA have been considered censored values as per survival analysis. Estimated Hazard ratios with their 95 % confidence intervals are provided.

## Results

Between January 1st 2004 and October 1st 2013, 155 RRMS patients (68.3 % females) who were treatment-naive (100 patients) or had been previously treated with IFNs (55 patients) were prescribed GA at the Neuroimmunology and MS Unit of La Paz University Hospital. Ages at GA treatment initiation ranged from 17 to 60 years of age, average 37(± 9) and were similar for both treatment groups (treatment-naive patients and patients treated previously with IFN). There was a tendency for a greater proportion of females in the treatment-naive group (73 % vs. 58.1 %, *P* = 0.073). Median EDSS at GA prescription was similar for both groups (Table 1).

**Table 1:** Characteristics of patients treated with glatiramer acetate (GA), January 2004 – October 2013

	Total patients (*n* = 155)	Treatment-naive (*n* = 100)	Previous treatment with interferon (*n* = 55)	*P*
Age: range, y	17 – 60	17 – 56	24 – 60	*p* = 0.3
Mean (± s.d.)	37 (± 9)	37 (± 9)	38 (± 8)	
Female sex, *n* (%)	106 (68.3)	73 (73.0)	32 (58.1)	*p* = 0.07
Geographical origin				
South Europe (Spain), *n* (%)	145 (93.5)	94 (94.0)	51 (92.7)	
Eastern Europe, *n* (%)		2 (2.0)	1 (1.8)	
North Africa, *n* (%)		2 (2.0)	1 (1.8)	
South America, *n* (%)		2 (2.0)	2 (3,6)	
Time to treatment^a^: Mean (± s.d.), y	5.2 (± 5.9)	2.7 (±5.5)	6.7 (± 5.7)	*p* < 0.001
EDSS at GA initiation (median [IQR])		1.5 [1–3]^b^	2 [1–3]^b^	*p* = 0.2

*y* years, *s.d* standard deviation, *GA* Glatiramer Acetate, *IQR* Interquartile Range

^a^Time to treatment = Average time-lapse from diagnosis to GA treatment initiation

^b^EDSS at GA initiation data availability of 55 %

**Fig. 1:**
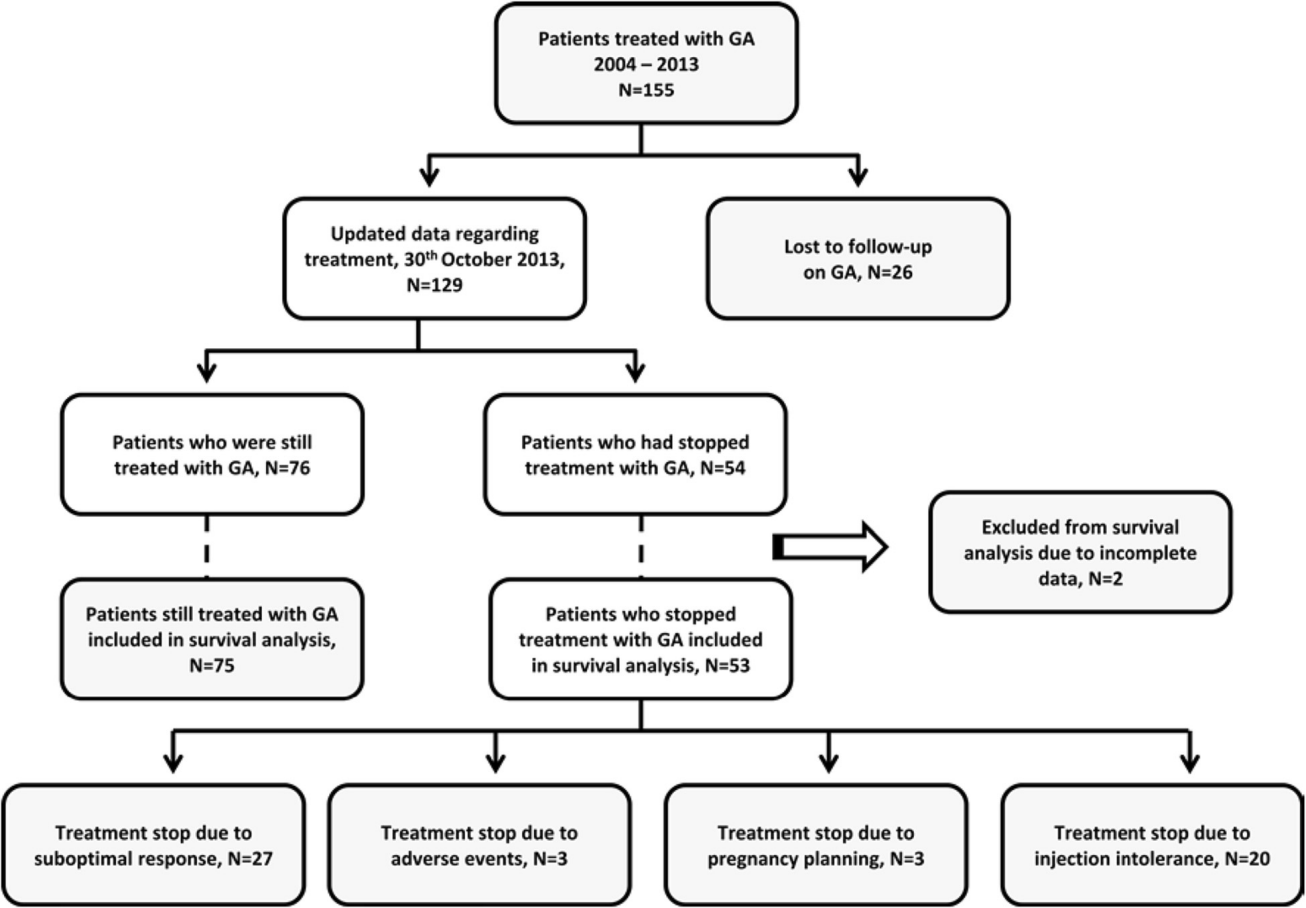
Relapsing Remitting Multiple Sclerosis (RRMS) patients treated with glatiramer acetate (GA), January 2004 – October 2013. Flow chart showing prescription of glatiramer acetate (GA) for Relapsing Remitting Multiple Sclerosis (RRMS) patients at the Neuroimmunology and MS Unit of La Paz University Hospital, Madrid, Spain, from January 2004 to October 2013. Patients lost to follow-up, those excluded from statistical analysis and reasons for stopping GA treatment are indicated

**Fig. 2:**
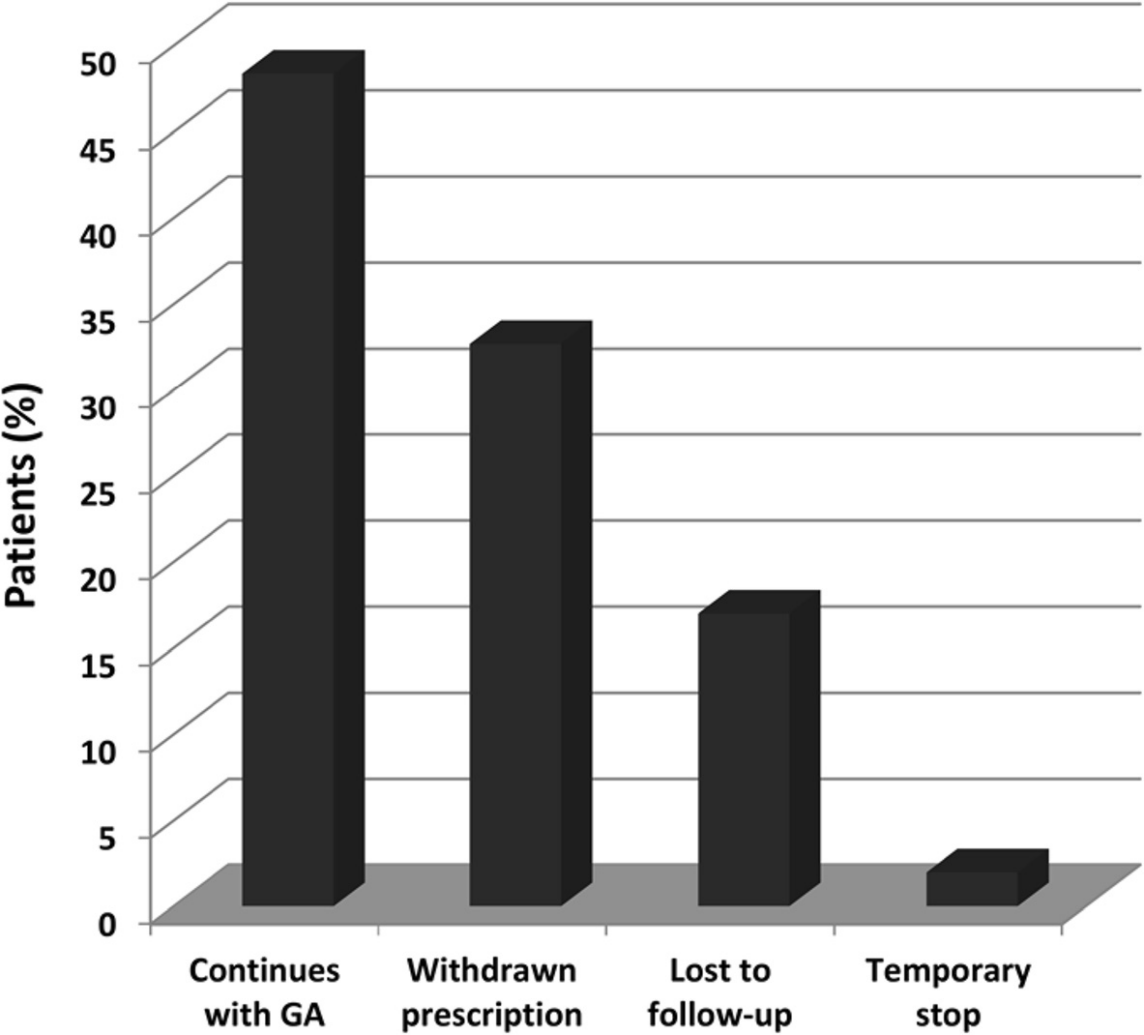
Proportion of patients in each treatment persistence group. Graph showing proportion of patients according to different glatiramer acetate (GA) treatment persistence groups at the Neuroimmunology and MS Unit of La Paz University Hospital on October 1st 2013

At the end of the study (Fig. 1), from the 155 patients, 76 patients (49.0 %) were still being treated with GA. Prescription had been withdrawn in 50 cases (32.3 %), 3 patients (1.9 %) had temporarily stopped GA for pregnancy planning and 26 patients (16.8 %) had been lost to follow-up (Fig. 2). From the 50 patients who stopped GA, all patients with previous IFN exposure escalated therapy to second line drugs, while those in the treatment-naive group followed a variety of pathways, including switching to second-line drugs (60.4 % patients), but also to IFN (29.2 %) and immunosuppressive drugs (4.2 %), as well as stopping treatment altogether following patients’ demands (4.2 %) or conversion to secondary progressive MS (2.1 %).

In our cohort, reasons for stopping GA treatment were: (a) lack of efficacy, including relapses (22 patients, 14.2 %), disability accrual (3 patients, 1.9 %) or evidence of disease activity on follow-up MRI (2 patients, 1.3 %); (b) injection intolerance (20 patients, 12.9 %): mainly injection pain and mild-moderate skin reactions such as redness or itching; (c) SAEs (3 patients, 1.9 %): injection associated unremitting chest pain, allergic reactions or severe local reactions such as marked lipoatrophy. Amongst patients who discontinued GA due to injection intolerance, most (*n* = 19 patients, 95 %) belonged to the treatment-naive group.

**Fig. 3:**
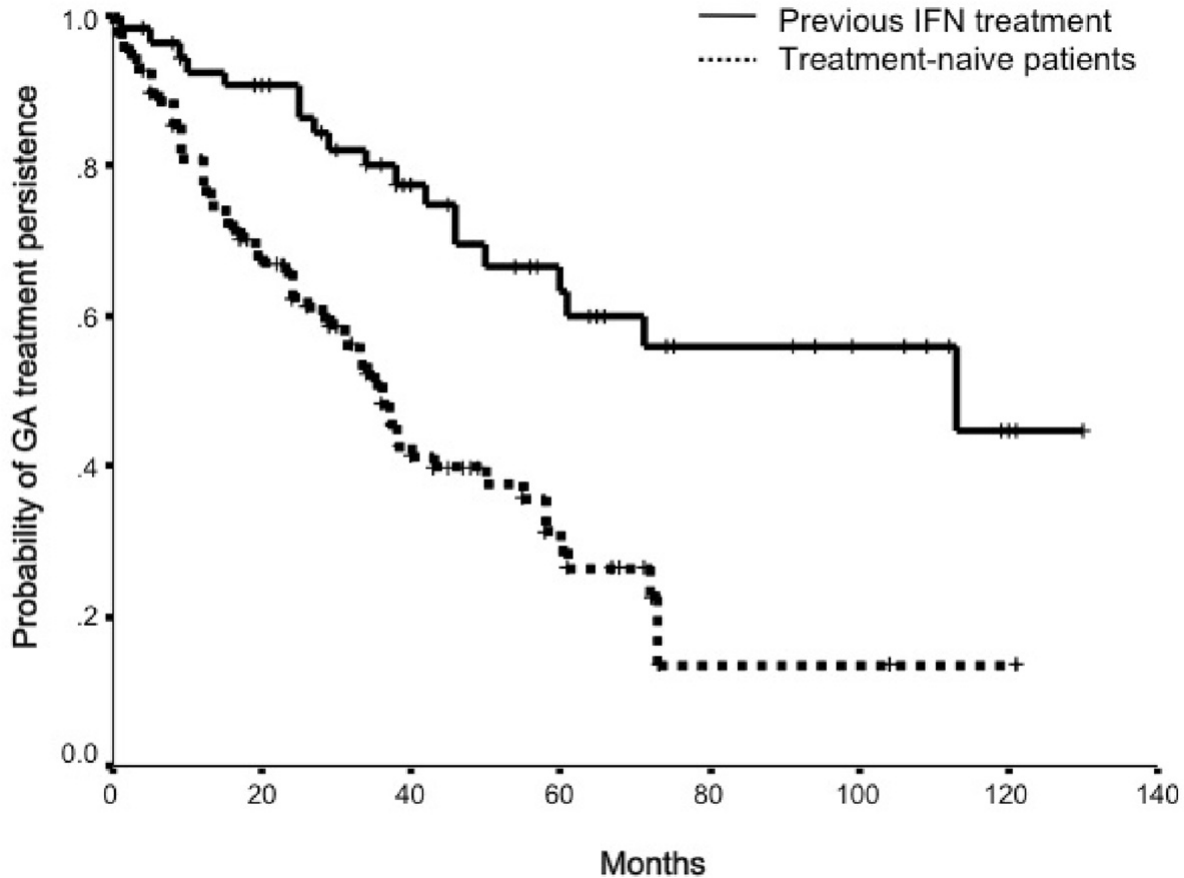
Treatment persistence on glatiramer acetate (GA) of treatment-naive patients and of patients treated previously with interferon (IFN). Kaplan-Meier survival curves showing treatment persistence on glatiramer acetate (GA) of treatment-naive patients and of patients previously treated with another disease modifying therapy (interferon)

Overall, patients in our cohort were treated with GA an average of 39 months (s.d 30.0). Median follow-up was 34 months. Six months after GA treatment initiation, probability of continuing on GA was 91.4 % (88.6 % for treatment-naive patients and 96.3 % for patients previously treated with IFN). Twelve months after treatment initiation, probability of continuing on GA was 82.5 % (76.8 % for treatment-naive patients and 92.5 % for patients previously treated with IFN). Two years after treatment initiation, probability of continuing on GA was 72.5 % (62.3 % for treatment-naive patients and 90.6 % for patients previously treated with IFN). Treatment-naive patients were found to be significantly more prone to discontinue GA than those with previous exposure to IFNs (Fig. 3). The risk of GA discontinuation was 2.8 [C.I. 95 %: 1.7 – 4.8] times greater for treatment-naive patients and this was hardly modified after adjusting for sex and age (3.0 [C.I. 95 %: 1.8 – 5.2]).

**Fig. 4:**
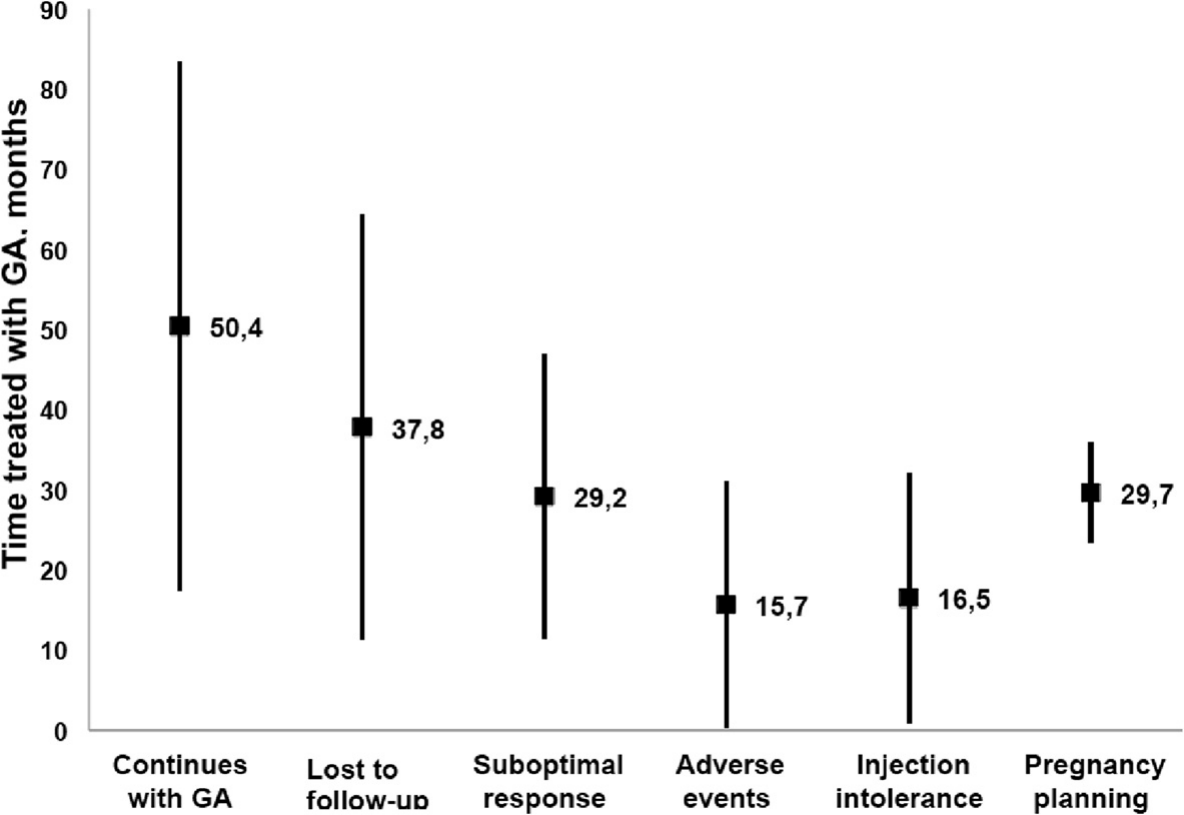
Average treatment-time with glatiramer acetate (GA) according to patient’s treatment persistence group. Graph showing average treatment-time (ATT) in months (±standard deviation) with glatiramer acetate (GA) at the Neuroimmunology and MS Unit of La Paz University Hospital between January 2004 and October 2013, according to patient’s GA treatment persistence category

Regarding time spent on GA, ATT for patients who persisted on GA at the end of the study was 50.4 months (s.d. 32.8). Patients who discontinued GA for lack of efficacy had an ATT of 29.2 months (s.d. 17.5), amongst patients discontinuing for SAEs, ATT was15.7 months (s.d. 15.1) and for injection site reactions ATT was 16.5 months (s.d. 20.3). Amongst patients lost to follow-up, ATT was 37.8 months (s.d. 26.3). ATT for patients discontinuing GA for pregnancy planning was 29.7 months (s.d. 6.0) (Fig. 4).

## Discussion

We present an independent, observational study analysing treatment persistence on glatiramer acetate (GA) in an MS Unit of a Spanish tertiary University Hospital. Little is known on how the use of previous DMDs affects treatment outcome. This is the second published study that aims to describe differences in treatment persistence with GA in treatment-naive patients with respect to patients previously treated with IFNs, and the first to do so in Europe.

Subcutaneous GA is a well-established treatment for RRMS. Although GA’s exact mechanism of action remains to be fully elucidated, key roles seem to be modulation of inflammatory response and neuroprotective effects [12]. Clinical trials and follow-up studies have shown that GA reduces clinical and radiological disease activity, proves more effective than placebo and shows at least similar efficacy to subcutaneous IFN β-1a and IFN β-1b [12–18]. In the literature, GA is described to be effective both in treatment-naive patients and in those switching from a previous DMD because of lack of efficacy or side effects [19].

The current paradigm is to start RRMS treatment with injectable DMDs as a first-line therapy and then advance into the therapeutic pyramid until the disease is effectively controlled. However, first-line treatment failure with IFNs may be due to the existence of neutralizing antibodies [12], poor tolerance or occurrence of adverse events, and may not reflect the need to escalate to second-line therapies such as Fingolimod or Natalizumab. Second-line drugs offer potentially greater efficacy, but are associated with an increased level of risk [3, 20], also costs sometimes need to be taken into account when evaluating treatment options. Thus, changes between first-line agents may be, in some cases, a suitable option.

In contrast to the situation in clinical trials, switching between DMDs is common in daily practice. Studying treatment use and response in ‘real-world’ clinical practice is relevant for the future care of RRMS. It is important to address questions not answered by clinical trials regarding not only efficacy, but also tolerability and treatment persistence [21]. Treatment persistence varies with country of residence [22]. Jokubaitis et al. compared treatment persistence on GA and IFN in Australian patients and report that, in their cohort, patients receiving IFNβ-1a as a first DMD persisted longer on treatment than those treated with GA as a first DMD [22]. Similarly, Kleinman et al. found lower discontinuation rates on IFN β than on GA in the US [23]. On the other hand, data from the REGARD clinical trial do not show significant differences in treatment persistence between IFNβ-1a and GA [24]. In the Australian cohort, higher EDSS and younger age at DMD initiation were predictive of treatment discontinuation, however in our cohort, adjusting for age hardly modified the relative risk of GA discontinuation. Another study on DMD use reported female sex as a predictive factor of treatment discontinuation [25], but this was not reproduced in the Australian study or in our cohort. Missing data do not allow us to conclude if EDSS is predictive of treatment discontinuation in our cohort.

With respect to efficacy, GA was useful in our ‘real world’ setting, with only 17.4 % of patients presenting breakthrough disease (relapses, progression of disability or MRI activity), which is lower than that of clinical trials and on the low end of reported breakthrough disease for clinical practice, which is of 18 – 30 % in 5 years for first-line DMDs [19]. This difference might be explained by clinical trials’ selective inclusion criteria that tend to include only highly active patients with a greater probability of break-through disease than that of “the average MS patient”. It may also result from underestimating real breakthrough activity, as patients who might have remained on GA despite disease activity are not included.

Regarding safety and tolerability, subcutaneous GA was generally well tolerated in clinical trials and extension studies, the most commonly reported adverse event being injection site reactions and mild vasodilation [12] (http://www.copaxone.com/about-copaxone/copaxone-safety-and-effectiveness, cited 2014 Mar 14). Our results agree with clinical trials regarding treatment safety, only 1.9 % patients developed relevant medical side-effects (injection associated unremitting chest pain, allergic reactions or severe local reactions such as marked lipoatrophy). However, our data show a relatively high treatment discontinuation rate (12.9 %) due to injection intolerance (mainly injection pain and mild-moderate skin reactions, including redness and itching), mostly amongst treatment-naive patients, and this was independent of gender. Along these lines, previous studies indicate that the lack of treatment tolerance is a major reason for DMD discontinuation [22, 26, 27].

The overall risk of GA treatment discontinuation in our cohort was almost 3 times greater for treatment-naive patients than for patients treated previously with IFN. Similarly, Jokubaitis et al. report lower treatment persistence on GA than on IFN only amongst treatment-naive patients and not when these drugs were used as second therapies [22]. We believe this might be influenced by non-naive patients being used to self-injections.

Patients who discontinue treatment with IFNs may do so because of side-effects or disease activity. With regard to risk-benefit ratios, MS disease activity of some patients may not justify assuming the potential risks of second-line drugs. This study argues in favour of incorporating GA in the MS therapeutic algorithm not only as an initial agent but as a second therapy for certain patients who discontinue treatment with IFNs. Further investigation regarding reasons for treatment discontinuation and comparisons between treatment-naive patients and switchers is required.

Limitations to our study include the retrospective design that limits study variables. The prevalence of IFN neutralizing antibodies was not consistently checked, treatment compliance was not well recorded and no questionnaire addressing patients’ perception of treatment was administered. Other limitations of this study are cohort size and loss to follow-up, with 26 patients (16.8 %) lost to follow-up on treatment with GA, however this seems reasonable taking into account the 10 year time-span of the study. This pilot study was designed as an exploratory study so we were not able to conduct certain sub-analysis. We were not able to eliminate potential bias by adjusting for disability which can influence treatment persistence [22, 25]. However, despite the limited sample size, significant results are obtained in treatment persistence between treatment-naive patients and patients treated previously with IFN. Regarding the clinical applicability of these results, the time frame must be taken into account as it may influence treatment persistence. In our cohort, patients who discontinued GA for lack of treatment efficacy were on GA for over two years (29.2 months (±17.5)). Patients underwent regular follow-up visits at least twice per year, so GA might be seen a useful initial treatment even if long-term response is suboptimal. However, patients might have remained on GA waiting for the arrival of new therapies. Between 2004 and 2013 progress in the treatment of MS was remarkable, with new second-line drugs being licensed. At the same time, MRI criteria for early identification of non-responders were developed and progressively incorporated into clinical practice. With regard to the future, the situation is one of an increasing complexity, as new oral drugs, available as first-line therapies, arrive [20, 28].

A recent review comparing comparing the efficacy of IFN with that of GA in RRMS concludes that, even if safety and efficacy parameters of both drugs are similar, patient needs such as different levels of tolerability need to be addressed and taken into account for treatment choice [29]. A prospective study with questionnaires specifically addressing subjective perception of treatments as well as treatment adherence, in both treatment-naive patients and switchers, might shed light on the differential treatment persistence.

## Conclusions

In summary, GA was safe and useful with low rates of serious adverse events, similarly to previously published data, and low rates of break-through disease. Injection intolerance proved a major limitation to GA use, mostly amongst treatment-naive patients. We conclude that prior DMD use seems to influence persistence on GA; patients with previous exposure to IFN presented a significantly lower probability of GA treatment discontinuation than treatment-naive patients.

## Abbreviations

ATT: Average treatment time
DMD: Disease modifying drug
GA: Glatiramer acetate
IFN: Interferon
MRI: Magnetic resonance imaging
MS: Multiple sclerosis
RRMS: Relapsing remitting multiple sclerosis
SAE: Serious adverse event
s.d: Standard deviation
